# Microfibrillar-associated protein 4 variation in symptomatic peripheral artery disease

**DOI:** 10.1186/s12967-018-1523-6

**Published:** 2018-06-08

**Authors:** Line Ea Hemstra, Anders Schlosser, Jes Sanddal Lindholt, Grith L. Sorensen

**Affiliations:** 1 Cancer and Inflammation Research, Department of Molecular Medicine, University of Southern Denmark, J.B. Winsløws Vej 25, 3rd Floor, Odense, Denmark; 2 Cardiovascular Research Unit, Viborg Hospital, Viborg, Denmark; 3 Center of Individualized Medicine in Arterial Diseases (CIMA), Department of Cardiothoracic and Vascular Surgery, Odense University Hospital, Odense, Denmark

**Keywords:** MFAP4, Peripheral artery disease, Mortality, Primary patency, Lower-extremity ischemia

## Abstract

**Background:**

Symptomatic peripheral artery disease (PAD) is an atherosclerotic occlusive disease affecting the lower extremities. The cause of symptomatic PAD is atherosclerosis, vascular dysfunctions, impaired angiogenesis and neointima formation. Microfibrillar-associated protein 4 (MFAP4) is an extracellular matrix protein, which is highly expressed in the heart and arteries and recently introduced as a potential mediator of pathological vascular remodeling and neointima formation. We aimed to investigate the relationship between serum MFAP4 (sMFAP4) and symptomatic PAD outcomes.

**Methods:**

A total of 286 PAD patients were analyzed if they had either intermittent claudication or critical lower-extremity ischemia (CLI) and followed for 7 years. The level of serum MFAP4 (sMFAP4) was measured by alphaLISA. Kaplan–Meier, Cox proportional hazard and logistic regression analysis were used to analyze the associations between upper tertile sMFAP4 and symptomatic PAD outcomes.

**Results:**

Patients with upper tertile sMFAP4 had an odds ratio (OR) of 2.65 (*p* < 0.001) for having CLI diagnosis. Further analysis indicated that patients with upper tertile sMFAP4 had a hazard ratio (HR) of 1.97 (*p* = 0.04) for cardiovascular death during the 7-years follow-up. However, analysis of 2-year primary patency showed that patients with upper tertile sMFAP4 had decreased risk of vascular occlusion after reconstructive surgery with HR of 0.15 (*p* = 0.02).

**Conclusions:**

sMFAP4 has potential as a prognostic marker for cardiovascular death, primary patency of reconstructed vessels and CLI diagnosis in symptomatic PAD patients. Confirmation of observations in larger cohorts is warranted.

**Electronic supplementary material:**

The online version of this article (10.1186/s12967-018-1523-6) contains supplementary material, which is available to authorized users.

## Background

Peripheral artery disease (PAD) is an atherosclerotic vascular disease that affects the blood vessels other than those in the coronary circulation. The cause of symptomatic PAD is atherosclerosis, vascular dysfunctions, impaired angiogenesis and neointima formation and the prevalence is increased with age and is a growing health problem worldwide. It has been estimated that approximately 10% of men between 65 and 74 years have PAD [1], which is associated with an increased mortality rate of 25–30% [2–5]. Studies of the future need for vascular surgery in PAD patients predicts that vascular procedures will increase from 1.39 million in 2015 to 1.6 million in the adult population by 2020 in US [6].

Symptomatic PAD can present as intermittent claudication (IC) or as critical lower-extremity ischemia (CLI). CLI is a life-treating syndrome and associated with high risk of amputation [7–10]. The proportion of diagnosed CLI patients is approximately 40% of hospitalized PAD patients [11]. The clinical manifestations of the disease are seen after the establishment of the flow-limiting atherosclerotic plaque and symptoms include intermittent claudication leg pain, rest pain, and ischemic ulcers. PAD patients further present with increased cardiovascular mortality [12] and increased functional decline compared to non-PAD [13]. The current treatment includes lifestyle changes, surgical revascularization [14] and medical therapy targeted at lowering atherosclerotic vascular diseases, which results in significant lowering of morbidity and mortality associated with PAD [15]. Consequently, targeted screening of at-risk patients using measurements of ankle–brachial index (ABI) is recommended [16]. Moreover, serum biomarkers have been used experimentally for the prediction and detection of PAD [17, 18], but although clear associations have been demonstrated, the overall clinical value related to patient outcomes remains unclear.

As such, identification of new biomarkers of symptomatic PAD is vital for early discovery and treatment of patients. Indication of biological processes, for instance, resulting in increased risk of needing vascular reconstruction or developing CLI has the potential to reduce the worsening of symptoms and thus the morbidity and mortality of symptomatic PAD patients. In the present study, we envisioned that microfibrillar-associated protein 4 (MFAP4) could serve as such a marker.

MFAP4 is an oligomeric extracellular matrix (ECM) protein belonging to the fibrinogen-related domain (FReD) super family, which also includes members such as angiopoietin and tenascin [19]. Besides the C-terminal FReD, the MFAP4 sequence harbors a N-terminal RGD sequence with integrin binding and activating capacity [20–22]. MFAP4 directly binds to collagen, elastin and fibrillin [23] and it is localized to ECM fibers in the heart and arteries throughout the body [24]. *Mfap4*-deficient mice have been generated but did not reveal any distinct role of MFAP4 in normal physiology, although the adult mice developed a mild airspace enlargement compatible with subtle elastic defects [25]. In contrast, in vivo studies have demonstrated direct roles for MFAP4 in vascular occlusive remodeling with delayed neointimal formation and blunted outward remodeling after carotid ligation in *Mfap4*-deficient mice [22]. MFAP4 is further found in a soluble form and serum MFAP4 (sMFAP4) levels are recognized to vary with cardiovascular disease [24] and pulmonary disease [26] although the strongest association is found with high sMFAP4 and liver cirrhosis [27–29]. In the present study, we hypothesized that sMFAP4 is positively associated with the symptomatic PAD outcomes cardiovascular disease (CVD) death, 2-year primary patency after reconstructive surgery, and CLI.

## Methods

### PAD patients

Five hundred and seven patients were consecutively included during November 1999 to December 2008. The patients had either intermittent claudication (IC) or critical lower-extremity ischemia (CLI) and were enrolled in the study at Vascular Surgery Department, Viborg Hospital, Denmark [30]. The PAD diagnosis was assessed during the clinical examination. Cases with acute lower limb ischemia were excluded. Serum samples (centrifuged at 2000×*g* for 10 min) were available for sMFAP4 measurements from 373 patients and we included data from all patients who were followed for 7 years. Patients with incomplete medical records of date of death or sMFAP4 measurements were thus excluded and a total of 286 (165 men and 121 women) patients with complete medical records were included in the final analyses.

A thorough medical history was recorded for all patients. Clinical information came from medical records or directly from patients. Patients were characterized as current smokers or as nonsmokers if they had previously been smoking or never had been smoking. Diabetes was defined by history of diabetes mellitus or the use of oral anti-diabetic drugs and/or insulin. Hypertension was diagnosed upon interview. Hypertension was defined by any history of hypertension with use of anti-hypertensive drugs for that purpose. Deaths from all causes were identified in the Danish National CPR-registry (the centralized civil register in Denmark). In addition, the patients were followed in the nationwide Danish registry of Vascular Surgery (Karbasen) for need for later surgery during the 7 years follow-up period and moreover for primary patency the first 2 years. Primary CVD causes of death were identified by International Classification of Disease-10 codes in the I01.0 through I99.9, and sudden death [31].

### AlphaLISA measurements of serum samples

The *AlphaLISA* immunoassay technique was used for detection of sMFAP4 in patient samples as previously described by Wulf-Johansson et al., detection is based on simultaneous target recognition by two different monoclonal anti-MFAP4 antibodies [24].

### Statistical methods

Continuous variables are presented as median with 95% confidence intervals. Continuous data are compared using Kruskal–Wallis test. Categorical variables are presented as n (proportion) and compared with the Chi square test. Kaplan–Meier and Cox proportional hazard analysis were used to analyze the risk of CVD death and all-cause mortality during the 7-year follow-up period and the risk of vascular occlusion after reconstructive surgery for patients divided into upper sMFAP4 tertile and lower/middle sMFAP4 tertiles. A log rank test was performed for each survival analysis, to test the differences between the groups. Logistic regression was used to analyze the risk of a CLI diagnosis and hazard ratios (HRs) with 95% confidence intervals were calculated for upper tertile sMFAP4 with age, sex, body mass index (BMI) and smoking as covariates. A *p* value < 0.05 was considered statistical significant. StataIC 14 (http://www.stata.com) was used to perform the analyses.

## Results

### Baseline observations

Baseline characteristics divided into lower/middle tertile sMFAP4 and upper tertile sMFAP4 are shown in Table 1. The lower/middle tertile sMFAP4 included 190 patients and the upper tertile sMFAP4 included 96 patients. Analysis of clinical variables between these two groups showed, that there was a significant difference when considering age (*p* = 0.01), male sex (*p* = 0.02), smoking (*p* = 0.03), CLI/IC diagnosis (*p* < 0.001), whereas there was a tendency for BMI-association to MFAP4 tertiles. The unadjusted baseline characteristics did not reveal increased proportions of patients with upper tertile sMFAP4 amongst those who died from cardiovascular causes during follow-up.

**Table 1:** Baseline characteristics divided into sMFAP4 lower/middle tertiles and upper tertile

	Lower tertile/middle tertile (n = 190)	Upper tertile (n = 96)	*p*-value
Age (years)	65.1 [63.2; 66]	69 [65.8; 69.6]	0.01
Male sex	119 (66%)	46 (48%)	0.02
Body mass index (kg/m^2^)	24.6 [24.8; 25.9]	24.2 [23.8; 25.5]	0.05
Smoking	119 (66%)	49 (51%)	0.03
Hypertension	96 (51%)	46 (48%)	0.68
Previous stroke	3 (2%)	2 (2%)	0.76
Diabetes mellitus	29 (15%)	17 (18%)	0.59
Statin	37 (19.5%)	17 (18%)	0.55
ACE inhibitors	34 (18%)	21 (22%)	0.60
Critical limb ischemia	76 (40%)	61 (64%)	< 0.001
Intermittent claudication	114 (60%)	35 (36%)	< 0.001
Reconstructive surgery at baseline and during follow up	61 (32%)	28 (29%)	0.61
Cardiovascular death during follow up	24 (13%)	19 (20%)	0.11

Continuous variables are presented as median (95% confidence interval) and compared using Kruskal–Wallis test. Categorical variables are presented as n (proportion) and compared with the Chi square test

Baseline characteristics divided into patients with and without vascular surgery are shown in Additional file 1: Table S1. Analysis of clinical variables between these two groups showed, that there was a significant difference when considering critical limb ischemia (CLI)/intermittent claudiacation (IC) diagnosis (*p* < 0.001).

### Endpoint distribution

**Fig. 1:**
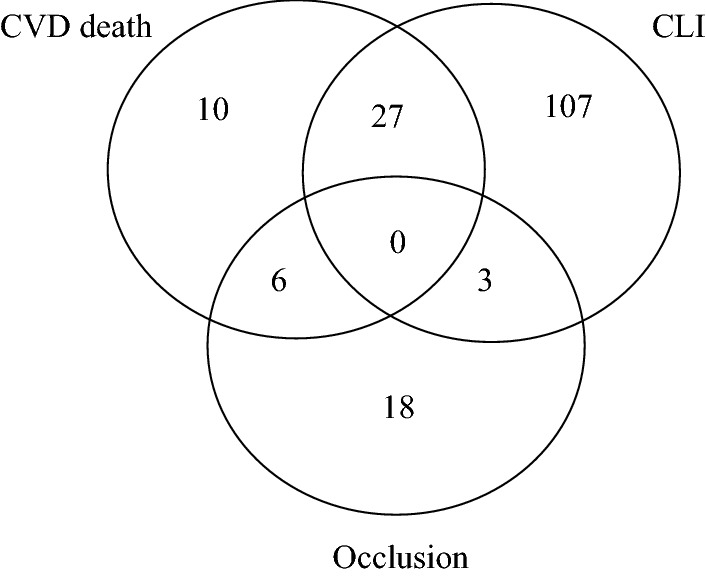
Distribution of patients, who were diagnosed with critical limb ischemia (CLI) at baseline, died of cardiovascular diseases (CVD death) or had a vascular occlusion after reconstructive surgery. Those patients who were diagnosed with intermittent claudication at baseline and did not suffer from CVD death or vascular occlusion are not included

Figure 1 shows the distribution and a limited overlap of patients with the clinical endpoints CVD death, vascular occlusion and the CLI diagnosis. The additional 115 patients who had IC diagnosis with no vascular occlusion or CVD death are not included in the figure.

### Symptomatic PAD patients with upper tertile sMFAP4 have significantly increased risk of CLI in adjusted analysis

Adjusted logistic regression analysis of patients with a CLI diagnosis (Table 2) showed that patients with upper tertile sMFAP4 have a significant OR of 2.65 (*p* < 0.001; 95% CI [1.56; 4.50]) for CLI diagnosis at baseline.

**Table 2:** Adjusted logistic regression analysis of risk of CLI diagnosis at baseline in symptomatic PAD patients

	Odds ratio	Std. err.	*p*-value	95% conf. interval
Upper tertile sMFAP4	2.65	0.71	< 0.001	1.56; 4.50
Age	1.02	0.01	0.201	0.99; 1.04
Male sex	1.53	0.40	0.103	0.92; 2.56
Body mass index (kg/m^2^)	1.02	0.04	0.588	0.95; 1.09
Smoking	0.72	0.19	0.204	0.43; 1.20

### Symptomatic PAD patients with upper tertile sMFAP4 have significantly increased risk of cardiovascular death during follow-up in adjusted analysis

**Fig. 2:**
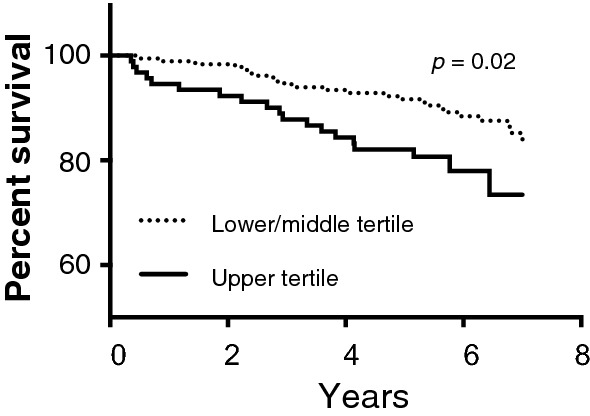
sMFAP4 upper tertile association with cardiovascular disease (CVD) death in symptomatic PAD patients during 7 years follow-up. A log rank test shows a significant difference (*p* = 0.02) between the two groups

Kaplan–Meier analysis (Fig. 2) and Cox regression (Additional file 1: Table S2) showed that patients with upper tertile sMFAP4 had a significant HR of 1.97 (*p* = 0.04; 95% CI [1.05; 3.70]) for cardiovascular death during the 7-year follow-up period. Log rank test of equality between the two groups showed a significant difference (*p* = 0.02) between patients with upper tertile sMFAP4 and lower/middle tertile sMFAP4 and the risk of cardiovascular death.

**Fig. 3:**
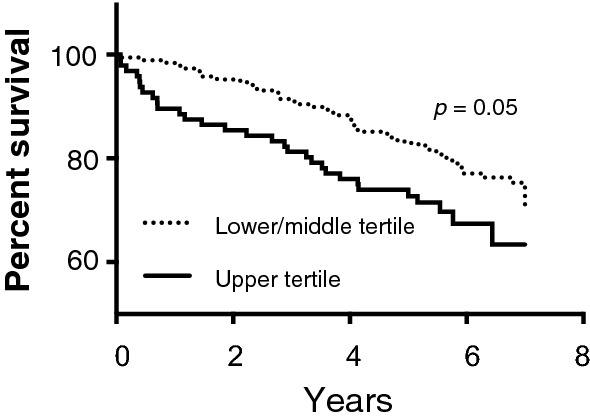
Kaplain-Meier survival estimate of all-cause mortality divided by sMFAP4 upper tertile. A log rank test for equality shows a p-value of 0.05 between the two groups

Kaplan–Meier analysis and Cox regression further showed that patients with upper tertile sMFAP4 had insignificantly (*p* = 0.07) increased all-cause mortality, with a borderline difference (*p* = 0.05) when comparing the two groups with a log rank test for equality (Fig. 3 and Additional file 1: Table S3).

### Symptomatic PAD patients with upper tertile sMFAP4 have significantly decreased risk of vascular occlusion in the 2-year period after reconstructive surgery

**Fig. 4:**
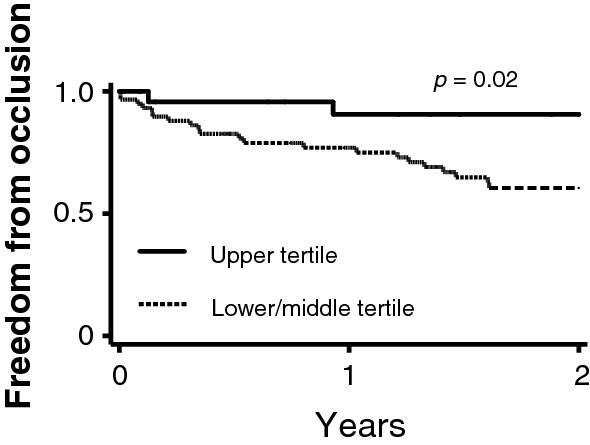
sMFAP4 upper tertile association with freedom from vascular occlusion during 2-year period after reconstructive surgery. A log rank test shows a significant (*p* = 0.02) difference between the two groups

Kaplan–Meier survival analysis (Fig. 4) was performed for occlusion of reconstructed vessels within an observation period of 2 years following reconstructive surgery. Log rank test for equality supported a significant (*p* = 0.02) difference between patients with upper tertile sMFAP4 and lower/middle tertile sMFAP4 when analyzing the risk of vascular occlusion. The data further showed that patients with upper tertile sMFAP4 had a decreased risk of occlusion of the reconstructed vessel with a HR of 0.15 (*p* = 0.02) (Additional file 1: Table S4).

## Discussion

The purpose of this study was to investigate whether sMFAP4 was positively associated with the following symptomatic PAD outcomes; CLI diagnosis, CVD death, and 2-year primary patency after vascular reconstruction. We observed a significant direct correlation between the upper sMFAP4 tertile and CLI. In addition, we observed that upper tertile sMFAP4 was significantly associated with CVD death in analyses adjusting for age, sex, smoking and BMI. Surprisingly, patients with upper tertile sMFAP4 had a significantly decreased risk of occlusion of their reconstructed vessel. The latter relationship may be explained by that there is a rather limited overlap between those patients with vascular occlusion after surgery, and CVD death and/or CLI in the present cohort (Fig. 1). Thus, a large proportion of patients with vascular occlusion after reconstructive surgery had IC (18/27) and no CVD death or CLI and sMFAP4 may be regulated differently in IC versus CLI or processes leading to CVD death.

This is to our knowledge the first observation that associates sMFAP4 to CLI, CVD death, and vascular occlusion after reconstructive surgery and the observation supports the hypothesis that sMFAP4 may serve as a prognostic marker in symptomatic PAD. As such, sMFAP4 has the potential to identify patients that are in risk of developing CLI or CVD death. However, it is currently unknown if high sMFAP4 develops as a consequence of disease and if a series of consecutive measurements therefore might be more informative than the present baseline measurement. Moreover, the study was not originally designed to test if inclusion of sMFAP4 into risk assessment based on ABI and Framingham risk scores would add value to a screening program.

Previous studies of clinical sMFAP4 variation showed that the level of sMFAP4 can be correlated to the other cardiovascular risk markers; fibulin-1, osteoprotegerin and ostepontin [24]. Basic studies performed by our group have shown that MFAP4 is involved in vascular smooth muscle hyperplasia, neointima formation and vascular remodeling in vivo [22]. The former studies in *Mfap4*-deficient mice have thus identified a mechanistic role of MFAP4 in vascular biology involved in vascular complications of PAD. Other observations have shown that both local MFAP4 expression and serum levels of MFAP4 are increased in liver fibrosis/cirrhosis [28, 29, 32] and moreover are moderately regulated in COPD [26]. On this basis, we do not suggest that MFAP4 is a disease-specific marker for symptomatic PAD.

A possible explanation of our present observations of the increased level of sMFAP4 in symptomatic PAD patients with the most severe outcomes may be that vascular bed remodeling and/or ischemia causes tissue disruption and release of ECM molecules, including MFAP4, into the circulation as seen previously [33]. The surprising association between the lowest sMFAP4 and increased risk of vascular occlusion after reconstructive surgery could indicate that certain events occurring in the vessels may contribute to destruction of MFAP4 as it is seen for matricellular proteins such as osteopontin [34, 35]. However, it was not examined if or to what degree sMFAP4 was degraded in the present study.

Limitation of the present study is furthermore that we do not have matched controls, that uncontrolled confounding and information bias might be present and that there was limited follow-up on CVD death in this study. Thus larger cohorts are warranted to confirm our observations. The strength of this study is that the prospective design has been used previously in several studies [30, 36–38]. Moreover, a relatively long duration of follow-up is present in this study.

## Conclusion

The present analysis shows that patients with upper tertile sMFAP4 have a significantly increased risk of CLI diagnosis and CVD death in adjusted analysis. Further analysis of risk of vascular occlusion within the first 2 years after reconstructive surgery shows a significantly decreased risk in patients with upper tertile sMFAP4. Validating analyses of these associations in larger cohorts are required to confirm that sMFAP4 may serve as a prognostic marker of CLI development and CVD death in symptomatic PAD.

## Additional file

**Additional file 1.** Additional tables.
