# Supplementary material for: Microfibrillar-associated protein 4 variation in symptomatic peripheral artery disease
Source: J Transl Med. 2018 Jun 8;16:159. doi: 10.1186/s12967-018-1523-6 (PMC5994031; doi:10.1186/s12967-018-1523-6)
Supplement: Supplementary file 1 — Additional file 1. Additional tables. [file 12967_2018_1523_MOESM1_ESM.docx]

**Additional material**

**Additional tables**

|  | Vascular surgery  (n= 89) | No surgery  (n = 197) | *p*-Value |
| --- | --- | --- | --- |
| Age (Years) | 67.6 [63.2; 68.5] | 67 [64.3; 67] | 0.30 |
| Sex (% men) | 52 (58%) | 113 (57%) | 0.87 |
| Body Mass Index | 24.5 [24.2; 26.32] | 24.5 [24.6; 25.6] | 0.55 |
| Smoking | 55 (62%) | 113 (57%) | 0.48 |
| Hypertension | 48 (54%) | 94 (48%) | 0.33 |
| Previous stroke | 1 (1%) | 4 (2%) | 0.93 |
| Critical limb ischemia | 21 (24%) | 81 (41%) | <0.001 |
| Intermittent Claudication | 68 (76%) | 116 (59%) | <0.001 |
| Diabetes mellitus | 20 (22%) | 26 (13%) | 0.05 |
| Statin | 21 (22%) | 33 (17%) | 0.17 |
| ACE inhibitors | 18 (20%) | 37 (19%) | 0.77 |
| Upper tertile of sMFAP4 | 28 (31%) | 68 (35%) | 0.61 |
| Cardiovascular death | 17 (19%) | 26 (13%) | 0.20 |

**Table S1. Baseline Characteristics divided by vascular surgery.** Continuous variables are presented as median [95% Confidence interval] and compared using Kruskal Wallis test. Categorical variables are presented as n (proportion) and compared with the chi square test.

|  | Haz. Ratio | Std. Err. | *p*-value | 95 % Conf. Interval |
| --- | --- | --- | --- | --- |
| Upper tertile of sMFAP4 | 1.79 | 0.63 | 0.04 | 1.05; 3.70 |
| Age | 1.06 | 0.02 | <0.01 | 1.02; 1.10 |
| Male sex | 1.61 | 0.54 | 0.16 | 0.83; 3.10 |
| Body mass index (kg/m^2^) | 1.02 | 0.04 | 0.60 | 0.94; 1.11 |
| Smoking | 0.66 | 0.22 | 0.21 | 0.35; 1.26 |

**Table S2.** **Adjusted Cox regression analysis of cardiovascular death in symptomatic PAD patients.**

|  | Haz. Ratio | Std. Err. | *p*-value | 95 % Conf. Interval | |
| --- | --- | --- | --- | --- | --- |
| Upper tertile of sMFAP4 | 1.55 | 0.38 | 0.07 | | 0.96; 2.50 |
| Age | 1.09 | 0.02 | <0.001 | | 1.05; 1.12 |
| Male sex | 1.73 | 0.43 | 0.03 | | 1.05; 2.83 |
| Body mass index (kg/m^2^) | 1.00 | 0.03 | 0.99 | | 0.94; 1.12 |
| Smoking | 1.01 | 0.24 | 0.979 | | 0.63; 1.60 |

**Table S3. Adjusted Cox regression analysis of all-cause mortality in symptomatic PAD patients**.

|  | Haz. Ratio | Std. Err. | *p*-value | 95 % Conf. Interval |
| --- | --- | --- | --- | --- |
| Upper tertile of sMFAP4 | 0.15 | 0.12 | 0.02 | 0.03; 0.70 |
| Age | 1.04 | 0.02 | 0.134 | 0.99; 1.08 |
| Male sex | 0.50 | 0.22 | 0.117 | 0.19; 1.20 |
| Body mass index (kg/m^2^) | 1.02 | 0.05 | 0.727 | 0.92; 1.13 |
| Smoking | 1.35 | 0.61 | 0.504 | 0.56; 3.30 |

**Table S4. Adjusted Cox regression analysis of primary patency after vascular reconstructive surgery in symptomatic PAD patients.**
